# AMP-independent activator of AMPK for treatment of mitochondrial disorders

**DOI:** 10.1371/journal.pone.0240517

**Published:** 2020-10-14

**Authors:** Tereza Moore, Rolando E. Yanes, Melissa A. Calton, Douglas Vollrath, Gregory M. Enns, Tina M. Cowan

**Affiliations:** 1 Department of Pathology, Stanford University, Palo Alto, CA, United States of America; 2 Department of Immunology and Rheumatology, Stanford University, Palo Alto, CA, United States of America; 3 Department of Genetics, Stanford University, Palo Alto, CA, United States of America; 4 Department of Pediatrics (Medical Genetics), Stanford University, Palo Alto, CA, United States of America; University of Florida, UNITED STATES

## Abstract

Mitochondrial diseases are a clinically heterogenous group of disorders caused by respiratory chain dysfunction and associated with progressive, multi-systemic phenotype. There is no effective treatment or cure, and no FDA-approved drug for treating mitochondrial disease. To identify and characterize potential therapeutic compounds, we developed an *in vitro* screening assay and identified a group of direct AMP-activated protein kinase (AMPK) activators originally developed for the treatment of diabetes and metabolic syndrome. Unlike previously investigated AMPK agonists such as AICAR, these compounds allosterically activate AMPK in an AMP-independent manner, thereby increasing specificity and decreasing pleiotropic effects. The direct AMPK activator PT1 significantly improved mitochondrial function in assays of cellular respiration, energy status, and cellular redox. PT1 also protected against retinal degeneration in a mouse model of photoreceptor degeneration associated with mitochondrial dysfunction and oxidative stress, further supporting the therapeutic potential of AMP-independent AMPK agonists in the treatment of mitochondrial disease.

## Introduction

Primary mitochondrial diseases encompass a clinically heterogeneous group arising from inherited deficiencies in the mitochondrial electron transport chain (ETC). These disorders commonly involve multiple organ systems and are characterized by prominent neurologic and myopathic features but can also affect a single tissue such as eye or muscle [1, 2]. Over half of affected patients are children, with the majority dying before adulthood. Molecular defects underlying mitochondrial disorders include pathogenic variants in either nuclear DNA (nDNA) or mitochondrial DNA (mtDNA) affecting the expression, replication or maintenance of mtDNA, as well as the function and formation of ETC complexes. Regardless of genetic etiology, respiratory chain abnormalities impair the transfer of electrons to molecular oxygen, leading to overproduction of reactive oxygen species (ROS) and decreased ATP synthesis [3]. The resulting imbalances disrupt signaling mechanisms responsible for metabolic homeostasis and lead to cellular damage, necrosis and apoptosis, and ultimately to the overt signs and symptoms of mitochondrial disease.

Despite this general understanding of disease mechanism, there still is no effective treatment or cure for mitochondrial disorders. Antioxidant supplements including nicotinamide, methylene blue, and *N*-acetylcysteine [4–8] have been shown to reduce levels of ROS in cell-based and mouse studies but have not shown clear benefit in patients with mitochondrial disorders [9–11]. Other potential antioxidant therapies including coenzyme Q_10_ (CoQ10), vitamin C, lipoic acid, EPI743 [Edison Pharmaceuticals]), KH176 [Khondrion BV]) and glutathione precursors (cysteine, *N*-acetylcysteine, RP103 [Raptor Pharmaceuticals]) [12, 13] have not shown consistent or conclusive therapeutic benefit.

We sought to identify additional therapeutic candidates for mitochondrial disease under the hypothesis that effective treatments should broadly address the consequences of mitochondrial disruption including both oxidative stress and energy depletion. Using a cell-based screening assay, we tested various compounds known to affect either ATP production or redox imbalance and identified AMP-independent activators of AMP-activated protein kinase (AMPK) as promising candidates. AMPK is the master regulator of intracellular homeostasis and central to the signaling pathways that maintain energy levels and oxidative balance [14, 15]. It is a heterotrimeric protein with a catalytic subunit, α, and two regulatory subunits, β and γ. Under conditions of increasing AMP:ATP ratio, AMP binds to the γ-subunit and allosterically activates AMPK through phosphorylation at Thr172 of the catalytic (α) subunit by upstream kinases LKB and Ca2+/calmodulin-dependent protein kinase β (CaMKKβ) [16]. The binding of AMP to the AMPK γ-subunit not only greatly enhances AMPK phosphorylation through upstream kinases, but also inhibits dephosphorylation of this activating phosphorylation by protein phosphatases, such as PP2A and PP2C [16]. Activated AMPK in turn upregulates ATP-producing pathways, including glycolysis and mitochondrial biogenesis, and downregulates energy-requiring pathways such as fatty acid synthesis and gluconeogenesis. AMPK also triggers the oxidative stress response by upregulating the expression of antioxidant enzymes including superoxide dismutase-2 (SOD2) and catalase, thereby addressing the multiple metabolic consequences of mitochondrial dysfunction.

The efficacy of AMPK agonists including metformin, resveratrol and 5-aminoimidazole-4-carboxamide ribonucleotide (AICAR) for treating mitochondrial disease has been explored with limited success and conflicting results [17–19]. Metformin and resveratrol activate AMPK indirectly by inhibiting the mitochondrial ETC and thereby increasing AMP, a mechanism that may be poorly suited for patients with mitochondrial disease stemming from primary ETC abnormalities. The AMP analog AICAR activates AMPK directly as AICAR 5'-monophosphate (ZMP) without altering ETC function or AMP levels, and has shown promise in studies of complex I-deficient fibroblasts with increased mitochondrial biogenesis, and complex IV-deficient mice with improved motor endurance [12, 18, 20]. However, because AICAR activates other AMP-regulated enzymes besides AMPK (e.g., the glycolytic enzyme phosphofructokinase), it also carries the risk of unwanted side effects (e.g., increased lactate production). AICAR is an endogenous intermediate in purine synthesis and also has a short half-life, making it an overall unsuitable therapeutic candidate [21].

Based on these studies, we searched for compounds that directly activate AMPK in an AMP-independent manner, i.e., by binding directly to the α or β subunit to promote AMPK phosphorylation. AMPK’s known role in glucose metabolism has prompted the development of a number of direct-acting AMPK activators in the diabetes field over the last decade. They were shown to be efficacious in rodent models of diabetes but have yet to progress to clinical trials, most likely a result of their poor pharmacological properties [22]. To determine whether preclinical studies would also demonstrate therapeutic potential in the treatment of mitochondrial disease, we tested these direct activators and found that they significantly improve cell viability in mitochondrial-deficient cultured fibroblasts. We show that this is accompanied by increased oxygen consumption and ATP concentration, reduced oxidative stress, and activation of AMPK downstream targets involved in mitochondrial biogenesis and oxidative stress response, and extend these findings to the SI mouse model of photoreceptor degeneration caused by oxidative stress. Our results demonstrate that AMP-independent allosteric activation of AMPK ameliorates both the energy depletion and oxidative stress arising from mitochondrial dysfunction and provide support for these compounds as attractive candidates for treating mitochondrial disease.

## Materials and methods

### Subjects

Fibroblasts previously obtained from four patients with mitochondrial disease and an aged-matched healthy control were used for this study. Samples were collected by 3mm punch biopsies from patient forearms. The patients harbored deficiencies in complex I (based on mito respiratory chain enzyme analysis), assembly factors of complex IV (SURF1: c.312_321del10insAT(p.L105X); c.845_846delCT (p.S282CfsX9) and COX10: c.809A>G (p.Y270C)/c.1145C>G (p.G268A), and the mitochondrial DNA polymerase (POLG: c.2243G>C (p.W748S); c.2542G>A (p.G848S). All samples were obtained with informed consent and approved by the Stanford IRB.

### Tissue culture

Fibroblast cell lines with less than 10 passages were maintained in DMEM medium containing 8.3mM glucose and supplemented with 10% fetal bovine serum (FBS)(Fisher Scientific), 1% Penicillin-Streptomycin (10,000U/mL)(Life Technologies), 1% glutaMAX (Life Technologies), 1% uridine (5mg/ml) and 1% pyruvate (11mg/ml) at 37°C, 5% CO2. Cells were treated with either DMSO or AMPK agonists, PT1 (Santa Cruz Biotechnology, Santa Cruz, CA; sc-361299), A-769662 (Apex Bio, Houston, TX; A3963), ZLN024 (Sigma-Aldrich, St. Louis, MO; SML0900), and C24 (Medchem Express, Princeton, NJ; HY-15840). Each drug was resuspended in DMSO at a stock concentration of 10 mM and diluted to a final concentration of 100uM in cell culture medium.

For cellular viability/morphology and ATP quantitation assays, 15x10^3^ cells/500ul media were seeded in quadruplicate on 24-well microtiter plates. The following day, the medium was removed, wells washed with PBS and replaced with glucose-free mitochondrial stressor media containing DMEM, 1% Penicillin-Streptomycin (10,000U/mL)(Life Technologies, South San Francisco, CA), 10% FCS (Fisher Scientific), 1mM Galactose (Sigma Aldrich, St. Louis, MO), and respiratory chain inhibitor (POLG and CI patient cells, 0.25uM rotenone; SURF1 patient cells 25uM sodium azide) with or without 100uM PT1 (Santa Cruz Biotechnology). Cells were then incubated for either 72 or 48hours and analyzed for growth/morphology and ATP levels, respectively.

For oxygen consumption and ROS assays, 15x10^4^/3ml media were seeded in triplicate on 6 well microtiter plates. The following day, the medium was removed, wells washed with PBS and replaced with DMEM medium containing 8.3mM glucose and supplemented with 10% fetal bovine serum (FBS)(Fisher Scientific), 1% Penicillin-Streptomycin (10,000U/mL)(Life Technologies), 1% glutaMAX (Life Technologies), 1% uridine (5mg/ml) and 1% pyruvate (11mg/ml) with or without 100uM PT1 (Santa Cruz Biotechnology). Following treatment with PT1, tissue cultures were analyzed for rate of oxygen consumption and levels of ROS.

### siRNA knockdown

siRNA for *PRKAA1* (Silencer® Select Pre-designed siRNA s101) (Thermo Fisher Scientific, Waltham, MA) and a negative control (Life Technologies) were incubated with Hiperfect transfection reagent (Qiagen, Germantown, MD) in media containing DMEM, 1% Penicillin-Streptomycin (10,000U/mL) (Life Technologies), 1% glutaMAX (Life Technologies) but no serum, and allowed to complex for 10 min at room temperature. The complex was then added to COX10-deficient patient tissue cultures in 6-well microtiter plates (final siRNA concentration of 10nM of each siRNA) and incubated for 72 hr at 37°C, 5% CO2. At the end of the incubation period, the cells were analyzed for levels of AMPK and pAMPK by western blot analysis, or incubated for six additional days with PT1 (or DMSO as untreated control) and assessed for viability by Calcein AM (described above).

### Assays

Cell growth was measured by a fluorometric method using Calcein AM (Anaspec, Fremont, CA). Mitochondrial stressor media was removed, wells washed with PBS and then incubated with 500ul/well of 800nM Calcein AM in PBS for 30 min at 37°C, 5% CO2. Cell viability was measured by values obtained using a 485 nm excitation with the Fluoroskan Ascent Microplate Fluorometer (Thermo Scientific).

Fibroblasts in tissue culture were visualized by phase-contrast microscopy with a Leica DM IRB microscope at ×10 magnification, and images were taken with the Hamamatsu ORCA-ER camera.

Cellular ATP content was measured by the CellTiter-Glo ATP Assay (Promega, San Luis Obispo, CA). Cells were lysed using RIPA buffer. The cell lysate was diluted 1:200 in PBS for a total volume of 50uL and then mixed with 50uL of CellTiter-Glo solution. The mixture was incubated for 10min at room temperature. After incubation, luminescence was measured using Turner Biosystems and ATP concentrations were calculated from a standard curve. ATP measurements were normalized to total protein measured using Quick Start Bradford 1X Dye Reagent (Bio-Rad, Hercules, CA; #500–0205).

ROS production was detected by CellRox Deep Red (Life Technologies). Cells were treated in the presence or absence of PT1 for 48 hours, trypsonized, washed in PBS and incubated with 5 μM CellROX Deep Red Reagent for 20 min at 37°C. Cells were then washed and resuspended in PBS. The signal was analyzed by flow cytometry using the BD LSRII (BD Bioscience, San Jose, CA). The results were analyzed using FlowJo software (Tree star Inc.).

### Oxygen consumption

Oxygen consumption rate (OCR) was measured using an XF96 extracellular flux analyzer (Seahorse Biosciences). Fibroblasts were seeded at 20x10^3^ cell/well in 100ul DMEM media containing 8.3mM glucose and supplemented with 10% fetal bovine serum (FBS)(Fisher Scientific), 1% Penicillin-Streptomycin (10,000U/mL)(Life Technologies), 1% glutaMAX (Life Technologies), 1% uridine (5mg/ml) and 1% pyruvate (11mg/ml) on an XF 96-well plate at 37°C, 5% CO2. The following day cells were washed with PBS and treated with fresh DMEM media with or without PT1 for 48 hours. After 48 hours of incubation, the media was replaced with 175ul unbuffered XF base DMEM medium (Fisher Scientific) with the same constituents as the DMEM medium and incubated at 37°C in a CO_2_-free incubator for 30min. Baseline OCR measurements were performed three times with four minutes of mixing and five minutes of measurement. After OCR baseline measurements, carbonylcyanide-p-triflouromethoxyphenylhydrazone (FCCP) was injected and the maximal OCR measured. Mitochondrial inhibitors oligomycin and antimycin was used to measure ATP-coupled and total mitochondrial respiration, respectively, while the residual respiration from antimycin inhibition construed non-mitochondrial respiration. ATP-coupled OCR was calculated by subtracting the OCR in the presence of oligomycin from basal OCR. Maximal OCR was calculated by subtracting the OCR in the presence of antimycin from those in the presence of FCCP. Following the experiment, total protein was measured using Quick Start Bradford 1X Dye Reagent (Bio-Rad #500–0205) and OCR measurements normalized to total protein.

### Simple western

Patient fibroblasts treated with PT1 and DMSO was lysed with RIPA buffer (Santa Cruz Biotechnolgy Cat#: sc-24948) and sonicated for complete cell lysis. Lysates were centrifuged at 4°C for 10 min at 10,000 RPMs and the supernatant was collected. Protein concentration was measured using Bradford Dye (BioRad Cat#: 5000205) and reading absorbance at wavelength 595 nm. Simple western was performed following manufacturer's instructions at a protein concentration of 0.4 ug/uL per sample. The samples were run on a Peggy Sue instrument by ProteinSimple Inc. The high sensitivity protocol was utilized by increasing the stacking loading time to 20 seconds, the sample loading time to 12 seconds, and the separation time to 45 min.

### qPCR

RNA was isolated from patient fibroblast that were treated with PT1 or DMSO. The RNA isolation was performed using RNeasy Micro Plus kit from Qiagen (Cat #: 74034) following manufacturer's instructions. Reverse transcription was done using VILO master mix (Cat#: 11755050). The generated cDNA was used for qPCR using power SYBR Green Master Mix (Cat#: 4367659) and analyzed by Applied Biosystems 7900HT. Primers available upon request.

### Mouse model with mitochondrial dysfunction

B6 mice were treated with 100mg/kg PT1 (or vehicle/DMSO) 24hrs and 12hrs prior to 35mg/kg sodium iodate (SI) (or vehicle/10% DMSO) treatment, then treated every 24hrs for 3 days post-SI. Both PT1 and SI were delivered by intraperitoneal (IP) injections and animals were phenotyped 3 days post-SI administration.

For retinal imaging microscopy, mice were anesthetized, and their pupils dilated using 1% atropine sulfate, 2.5% phenylephrine hydrochloride, and 0.5% proparacaine hydrochloride. Funduscopy was performed using the Micron III small animal retinal imaging AD camera (Phoenix Research Laboratories, INC). ONL thickness was measured from H&E sections using ImageJ. Three sections per animal were analyzed by taking seven distance measurements per section and averaged. Retinal function was evaluated by recording of dark-adapted ERG responses (Espion E2 System, Diagnosys LLC). Mice were dark adapted overnight before ERG recording, and all procedures were performed in the dark or under dim red light. Mice were anesthetized and their pupils dilated as described above. For the ERG recordings, electrodes were placed on the center of cornea. A ground needle electrode was placed in the base of the tail, and reference needle electrode was placed subdermally between the eyes. The a-wave amplitude was measured from the baseline to the trough of the a-wave, and b-wave amplitude was measured from the trough of the a-wave to the peak of the b-wave.

### Statistics

Comparisons between groups were made using a 2-tailed, unpaired Student’s t test. Data are presented as the mean ± SEM, and results were considered statistically significant if P was less than 0.05.

### Study approval

All procedures performed in this study were in compliance with the *Association for Research in Vision and Ophthalmology Statement for the use of Animals in Ophthalmic and Visual Research* and were approved by Stanford University Administrative Panel on Laboratory Animal Care.

## Results

### AMP-independent AMPK agonists improve cell viability of patient derived mitochondrial-defective cells

**Fig 1:**
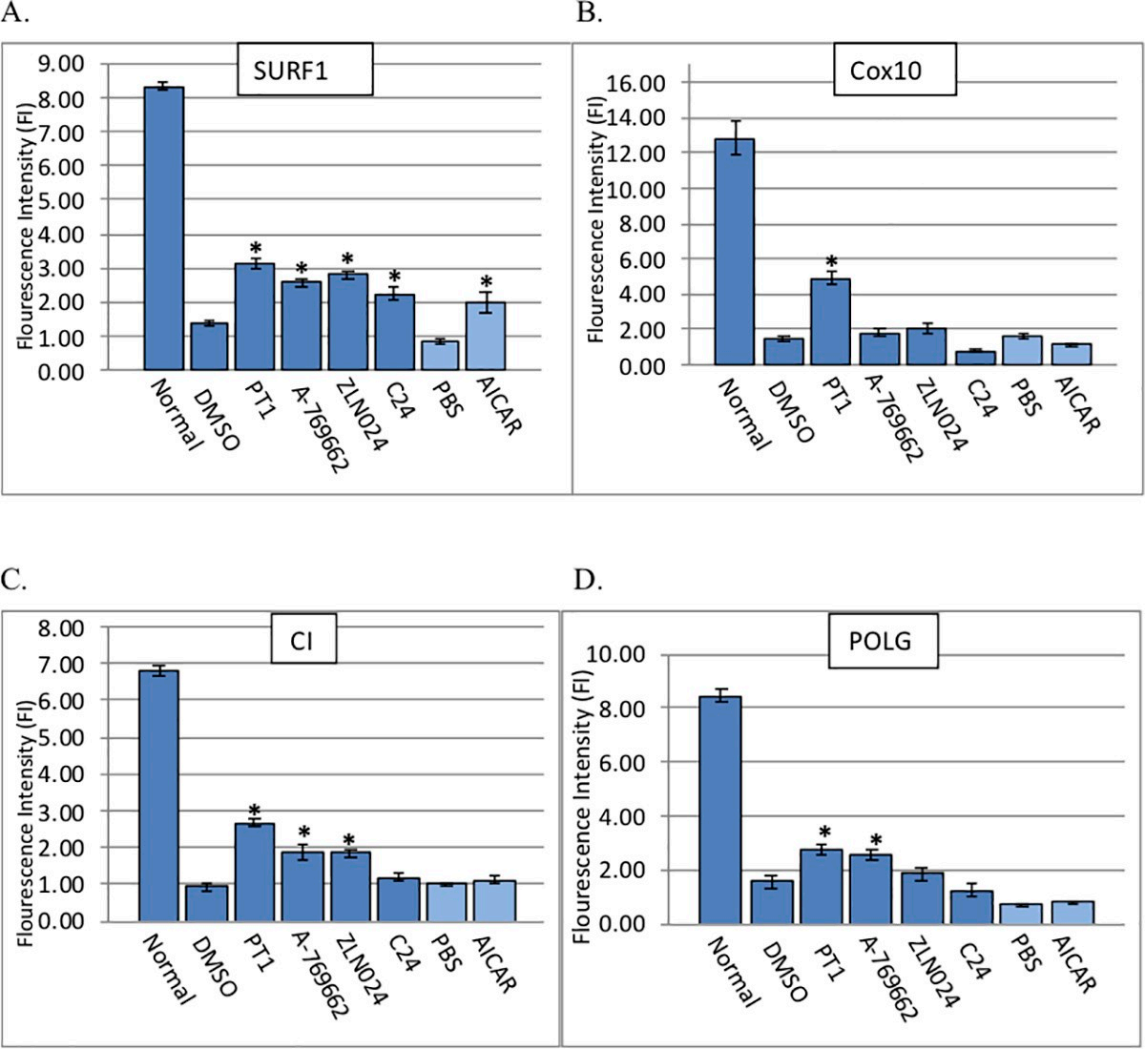
Effect of AMPK agonists on cell viability. Mitochondrial-deficient patient fibroblasts were treated with AMPK agonists A-769662, PT1, C24, ZLN024, AICAR, DMSO (vehicle), or PBS (untreated control). Cell lines are from patients with a defect in (**A**) SURF1, (**B**) COX10, (**C**) CI, and (**D**) POLG. Viability was assessed by calcein AM fluorescence intensity. Experiments were performed in triplicate on at least three separate occasions and also included fibroblasts from a healthy control (“Normal”). Bars represent standard error of the mean. Asterisks represent statistical significance (*p<0.05).

Initial studies were designed to test the hypothesis that AMPK is a potential therapeutic target for treating mitochondrial disease. We focused on compounds that directly bind to and activate AMPK without any significant change in cellular ATP, ADP or AMP levels. Compounds were screened by adding them to fibroblast cultures from a patient with SURF1 deficiency, defective in mitochondrial Complex IV assembly, and measuring the effects on cell viability by calcein‐AM fluorescence. The four compounds we tested, PT1, A-769662, ZLN024, and C24, bypass the regulatory gamma subunit and allosterically activate AMPK by binding directly with the alpha (PT1, ZLN024, and C24) or beta (A-769662) subunits [23–28]. The AMP analog AICAR, previously shown to improve mitochondrial function and viability of mitochondrial-defective patient cells [18], was used as a positive control. All of the direct AMPK agonists increased SURF1-deficient cell viability 35–55% over cells treated with DMSO (vehicle) alone (Fig 1A).

**Fig 2:**
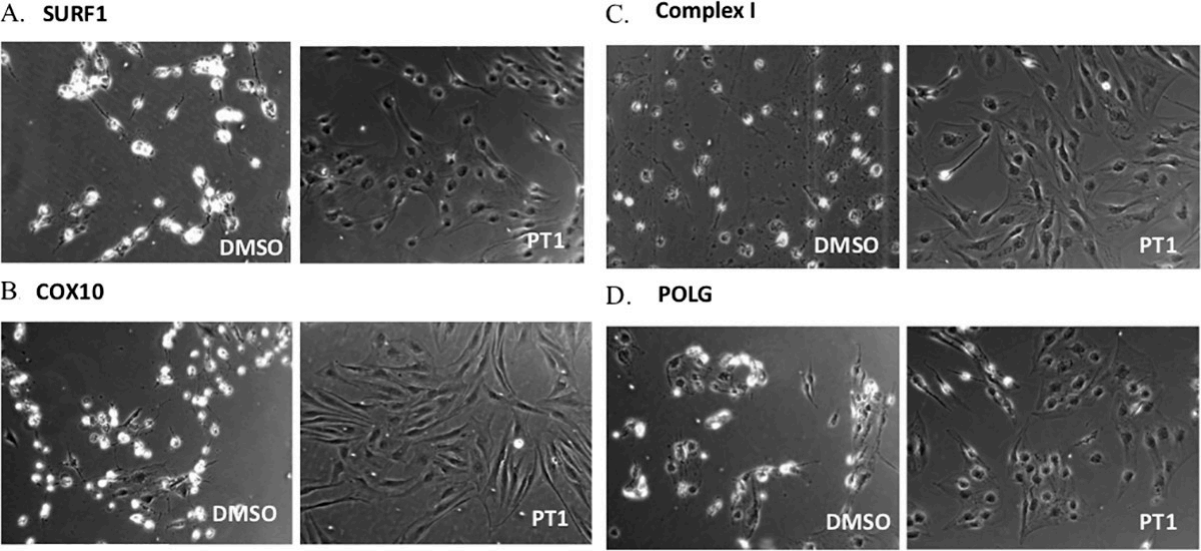
Effect of PT1 treatment on cell morphology. Mitochondrial-deficient patient fibroblasts were treated with the AMPK agonist PT1 or DMSO (vehicle) and morphology was visualized by phase contrast microscopy. Cell lines are from patients with a defect in (**A**) SURF1, (**B**) COX10, (**C**) CI, and (**D**) POLG.

We extended these studies to additional mitochondrial disorders representing a range of disruptions of the ETC and mtDNA replication machinery. Using the same direct AMPK activators as above, we treated fibroblasts from patients with deficiencies of mitochondrial complex I (CI), heme A:farnesyltransferase cytochrome c oxidase assembly factor (COX10), and mitochondrial DNA polymerase gamma (POLG). One compound, PT1, consistently improved viability in all mutant lines (Fig 1B–1D), with variable effects seen from the other compounds: A-769662 improved survival in CI and POLG-deficient cells, ZLN024 improved survival in CI-deficient cells, and C24 improved survival in SURF1-deficient cells but not in any of the other lines. In addition, AICAR improved viability only in SURF1-deficient cells. Taken together, results demonstrate that direct AMPK activators improved cell viability of mitochondrial-defective patient cells, with PT1 showing the most consistent effect across a range of mitochondrial disease etiologies. Similarly, PT1 treatment improved mutant cell morphology as shown by a flattened, elongated shape typical of normal fibroblasts in treated cells, as opposed to the more rounded, dispersed appearance of untreated cells (i.e., cells treated with DMSO alone) (Fig 2A–2D). Taken together, results indicate that AMP-independent activation of AMPK may be more effective in restoring mitochondrial function than the AMP-dependent mechanisms previously investigated. Because the most striking effects were seen with PT1, this compound became the focus of further investigation.

### PT1 improves viability of mitochondrial-defective patient cells through activation of AMPK

**Fig 3:**
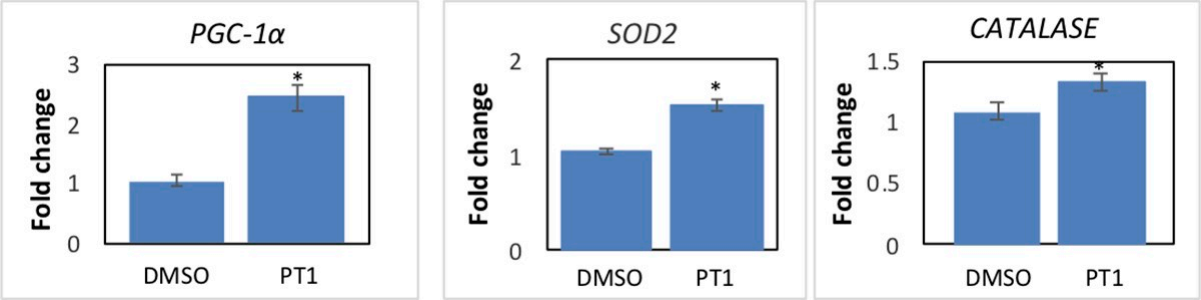
Effect of PT1 on downstream targets involved in mitochondrial biogenesis and oxidative stress. SURF1-deficient patient fibroblasts were treated with PT1 or DMSO (vehicle) for 24 hours and expression of *PGC-1* and *catalase/SOD2* RNA was evaluated by qPCR (n = 3). Results are normalized to actin. Asterisks represent statistical significance (*p<0.05).

To investigate the relationship between PT1 and AMPK activation, we characterized the effects downstream of this activation by measuring RNA expression in three target genes of AMPK, peroxisome proliferator-activated receptor gamma coactivator 1-alpha (*PGC-1α*), a master regulator of mitochondrial biogenesis, and catalase (*CAT*) and manganese-superoxide dismutase (*SOD2*), two of the main antioxidant genes regulated by AMPK [29]. SURF1-deficient cells were treated with PT1 or DMSO (vehicle) for 24 hours and RNA levels were evaluated by qPCR. Results demonstrated a 2.4-fold increase in *PGC-1α*, and 1.3-fold and 1.5-fold increases in SOD2 and catalase respectively in treated vs. untreated cells (Fig 3), supporting the critical roles of both mitochondrial biogenesis (through *PGC-1α* expression) and oxidative stress mitigation (*SOD2* and *CAT*) to the PT1 response in mitochondrial-deficient cells.

**Fig 4:**
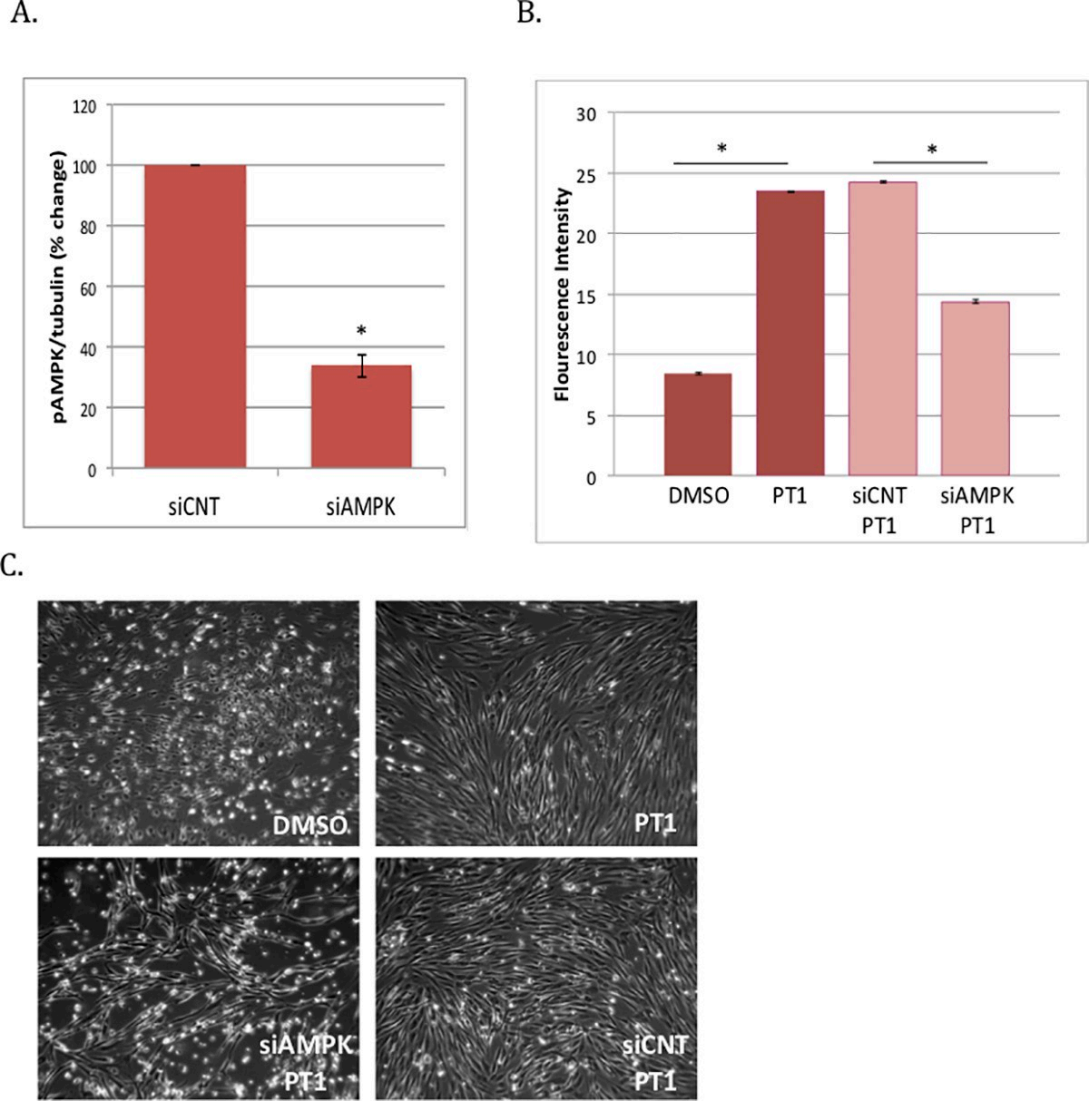
siRNA-mediated knockdown of *PRKAA1* (AMPK). COX10-deficient patient fibroblasts were transfected with AMPK-specific siRNA (siAMPK) or control siRNA (siCNT). Following transfection, cells were (**A**) evaluated for protein expression by western blotting, or (**B**) treated with PT1 or DMSO (vehicle) for an additional 6 days and then evaluated for viability by calcein-AM fluorescence assay and (**C**) morphology by phase contrast microscopy.

We next used an siRNA-mediated knockdown strategy to specifically inhibit *AMPK* gene expression and directly demonstrate the dependence of AMPK to the PT1 response. We treated COX10-deficient fibroblasts with PT1 and either siAMPK, a small interfering RNA that targets *PRKAA1*, the gene encoding the AMPK alpha subunit, or siCNT, a nonspecific siRNA sequence. Following treatment, cells were evaluated for protein expression by simple western analysis (Fig 4A), viability by calcein-AM fluorescence assay (Fig 4B), and morphology by phase contrast microscopy (Fig 4C). PT1 treatment together with selective knockdown of AMPK (PT1+siAMPK) resulted in a 63% decrease in pAMPK levels and 41% decrease in cell viability compared to control conditions (PT1+siCNT), consistent with the idea that cellular response to PT1 is mediated by AMPK activation.

### PT1 improves cellular respiration, increases ATP and decreases ROS in mitochondrial-defective patient cells

**Fig 5:**
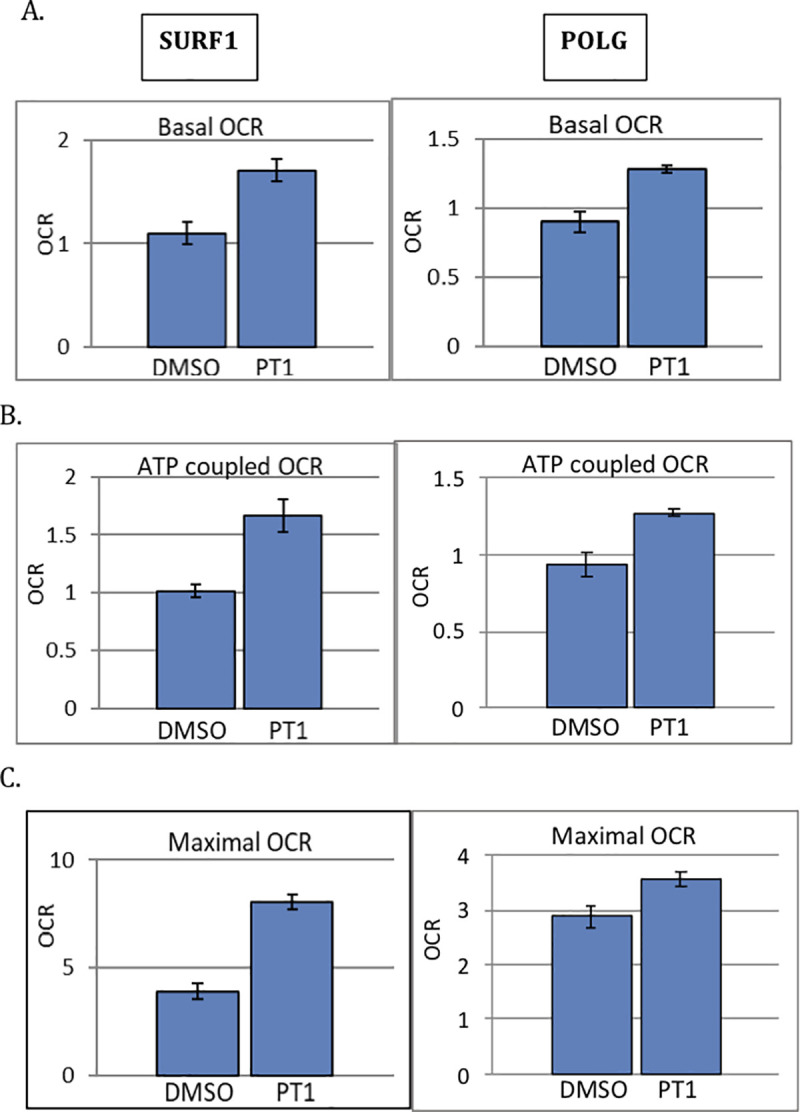
Effect of PT1 on cellular respiration in mitochondrial-defective patient cells. SURF1 and POLG-deficient patient cells were treated with PT1 or DMSO (vehicle) for 48 hrs and (**A**) basal, (**B**) ATP-coupled, and (**C**) maximal OCR were measured by the XF96 analyzer. All experiments were done in triplicate. Maximal OCR was measured after FCCP injection and ATP-coupled OCR following oligomycin injection. Results are normalized to total protein concentration. Data are represented as the mean ± standard error of the mean. *p<0.05.

**Fig 6:**
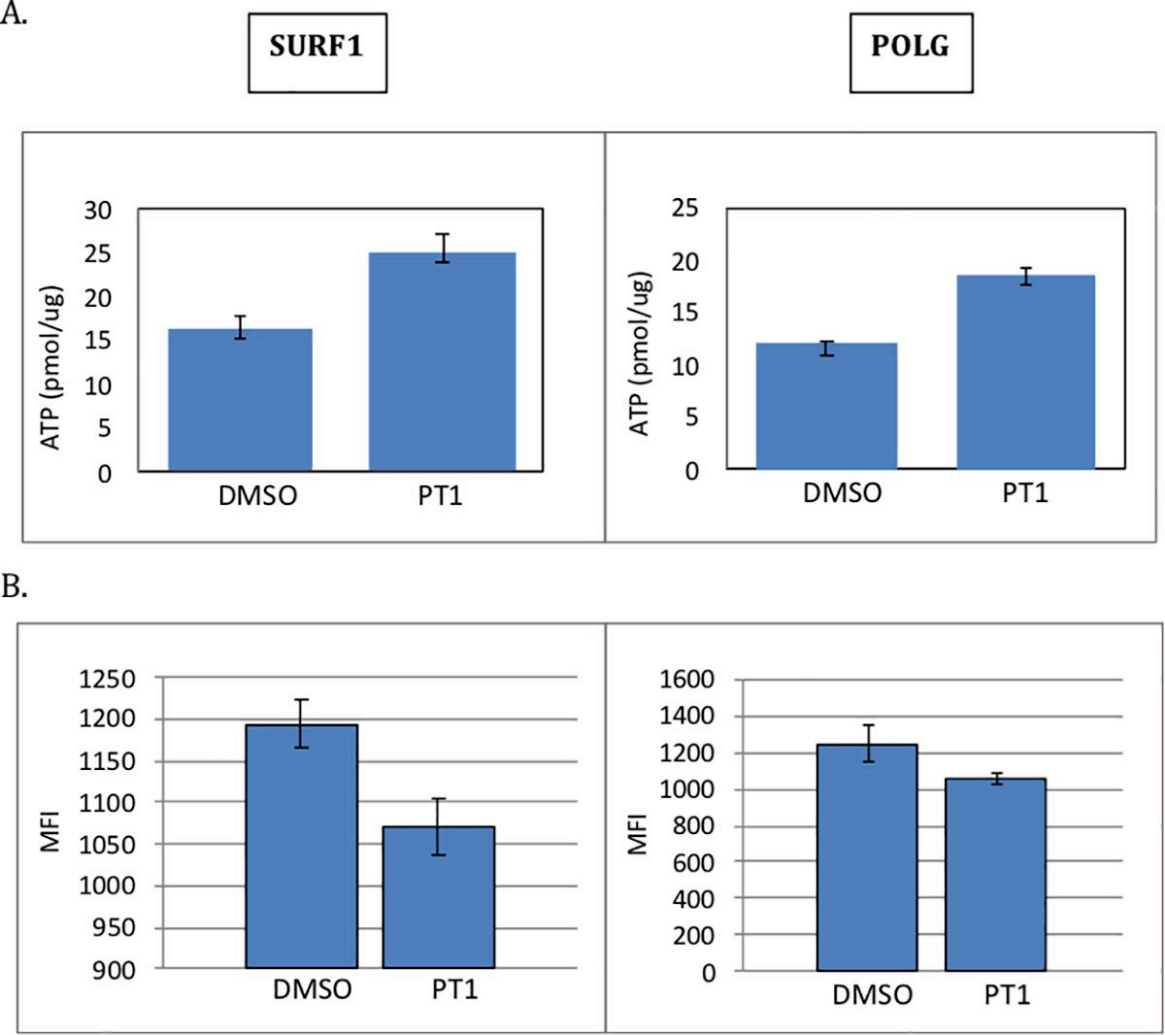
Effect of PT1 on ATP content and ROS in mitochondrial-defective patient cells. SURF1 and POLG-deficient patient cells were supplemented with PT1 or DMSO (vehicle) for 48 hrs. (**A**) ATP levels were measured by CellTiter-Glo ATP Assay, and results were normalized to total protein concentration. (**B**) ROS were levels measured by the CellROX Deep Red Flow Cytometry Assay. All experiments were done in triplicate. Data are represented as the mean ± standard error of the mean. *p<0.05.

Having demonstrated a direct relationship between PT1 and activation of AMPK and its downstream targets, we next tested the functional effects of PT1 treatment by monitoring cellular respiration and levels of ATP and reactive oxygen species (ROS), all known to be disrupted in mitochondrial patients due to diminished ETC function [30]. We reasoned that PT1-mediated mitochondrial biogenesis would improve respiration in patient cells with mitochondrial damage caused by accumulating reactive oxygen species (ROS). We assessed mitochondrial respiration using a Seahorse platform measuring oxygen consumption rates (OCR) at baseline (basal) or following treatment with oligomycin (ATP-coupled) or the uncoupler FCCP (maximal). Using this design, we evaluated the response to PT1 using cells from patients with SURF1 and POLG-deficiency, two distinct causes of mitochondrial disease. Both mutant cell lines demonstrated a roughly 30% improvement in basal respiration with PT1 treatment (Fig 5A). Furthermore, ATP-coupled respiration, or the fraction of basal mitochondrial oxygen consumption used for ATP synthesis, improved by 40% in SURF1 and 30% in POLG (Fig 5B). Maximal respiration, reflecting overall capacity to respond to increased ATP demand, improved in both SURF1 and POLG cells, with a 50% and 20% increase, respectively (Fig 5C). Consistent with these improvements in respiration, ATP content increased with treatment by 35% in SURF1 and 36% in POLG patient cells, and ROS levels decreased by 10% and 15%, respectively (Fig 6A and 6B), supporting the idea that AMPK activation through an AMP-independent rather than dependent mechanism may be more suitable for improving mitochondrial dysfunction.

### PT1 attenuates retinal degeneration in a mouse model of oxidative stress and mitochondrial dysfunction

**Fig 7:**
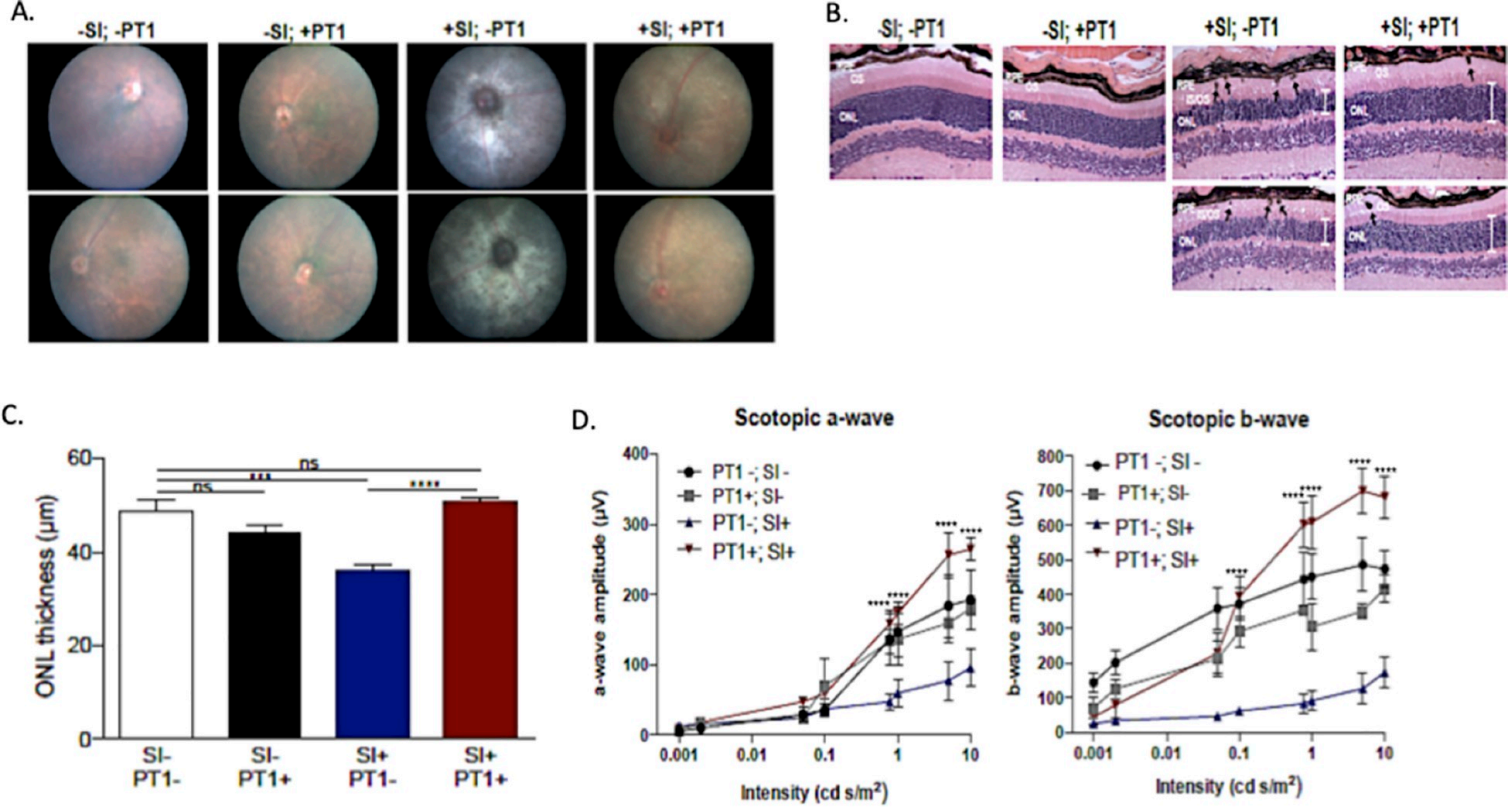
PT1 attenuates retinal degeneration in a mouse model of mitochondrial dysfunction. Sodium iodate-treated (+SI) or untreated (-SI) mice were treated with PT1 or DMSO (vehicle). The four groups (n = 5 mice per group) were evaluated by (**A**) fundoscopy; (**B**) H&E staining of retinal sections showing the outer nuclear layer (ONL; white bars), pigmented cells (black arrows), and photoreceptor inner outer segment (IS/OS) morphology; (**C**) quantitative assessment of ONL thickness; and (**D**) functional evaluation by ERG with measurements of the dark-adapted photoreceptor response (scotopic a-wave) and inner retinal response (scotopic b-wave). ERG data represent 3–5 mice per group and are plotted as mean ± SEM. *P<0.05; **P<0.01; ***P<0.001; ****P<0.0001.

To extend these studies *in vivo*, we evaluated the PT1 response in a chemically induced mouse model of mitochondrial dysfunction with retinal degeneration [31, 32]. Sodium iodate (SI) is a toxin that selectively induces oxidative stress and mitochondrial dysfunction in retinal pigment epithelium (RPE), ultimately resulting in retinal degeneration. SI-injected C57BL/6 (B6) mice showed characteristic signs of retinal degeneration, including white spots observed by funduscopy, thinning of the photoreceptor outer nuclear level (ONL), disorganization of the photoreceptor outer/inner segments (IS/OS), and presence of pigmented cells in the OS layer by H&E staining. Functional studies of SI-treated animals by electroretinography (ERG) demonstrated decreased dark-adapted (scotopic) electrical responses. In contrast, SI-treated mice who also received PT1 showed improved signs including decreased retinal depigmentation, decreased photoreceptor ONL) thinning, and decreased accumulation of pigmented cells in the OS layer (Fig 7A–7C). Additionally, dark-adapted ERG amplitudes (both scotopic a-wave and b-wave) were significantly higher in PT1 treated mice challenged with SI compared to SI-treated mice without PT1, demonstrating that PT1 treatment can protect retinal function from an acute oxidative insult (Fig 7D).

## Discussion

Despite progress in understanding the underlying mechanisms for mitochondrial disease, there are still no FDA approved therapies. One attractive therapeutic target, AMPK, responds to low-energy states by upregulating cellular uptake of oxidative substrates (glucose and fatty acids), energy-producing pathways (fatty acid oxidation and mitochondrial biogenesis), and the expression of antioxidant enzymes. We hypothesized that patients with mitochondrial disease would similarly benefit from AMPK activation as a means of overcoming reduced energy production and oxidative stress associated with ETC dysfunction. Our studies focus on direct agonists that allosterically activate AMPK through an AMP-independent mechanism, thus bypassing the regulatory inputs from modulating levels of AMP. We demonstrate that direct-acting AMPK agonists improve mitochondrial function, increase energy levels, and reduce oxidative stress, resulting in an overall improvement in cellular viability and tissue health.

Previous reports of AMP-dependent AMPK activators in mitochondrial disease provide variable support for AMPK as a therapeutic target. Consistent with these studies, we observed improved viability of mitochondrial defective cells following AICAR treatment, but the degree of recovery varied greatly between experiments and was not accompanied by improvement in cellular respiration [18]. Studies of other conventional AMP-dependent AMPK activators (e.g., metformin, resveratrol) as mitochondrial drug candidates yielded variable results with no clear efficacy [17–19]. Because these compounds share a common mechanism of ETC inhibition leading to AMP-dependent AMPK activation, they may be poorly suited for mitochondrial patients and were not pursued further in our studies. In general, AMP-dependent activators of AMPK can cause side effects such as the activation of other AMP-regulated enzymes, in some cases even exacerbating the effects of mitochondrial dysfunction.

Our studies show that compared to conventional AMPK activators working through AMP-dependent mechanisms, AMP-independent allosteric activators of AMPK may be more effective in treating patients with mitochondrial disease. Here we focus on the direct AMPK activators PT1, A-769662, ZLN024, and C24, originally identified in high throughput screens for small molecule activators of AMPK to treat diabetes, obesity and metabolic syndrome. Unlike AMP-dependent agonists, these compounds were shown to allosterically activate AMPK by binding directly to the α or β subunit, rather than the AMP-binding γ subunit, to promote AMPK phosphorylation [23–28]. PT1, A-769662, and ZLN024 significantly improved the viability of cells from patients with distinct underlying sites of mitochondrial impairment (complex I, complex IV, and mtDNA replication). Of the four compounds tested, C24 was the least effective and improved viability in only one patient cell line. The limitations of C24 may stem from its dual mechanisms of AMPK regulation via AMP-independent allosteric activation as well as AMP-dependent activation through ETC inhibition, the latter being a counter-effective mechanism in the context of mitochondrial dysfunction [33].

Among the AMP-independent AMPK activators tested, PT1 showed the most consistent and significant effects across the genetically distinct cell lines. Treatment with PT1 improved cell viability and morphology that was accompanied by increased cellular respiration and ATP and decreased ROS. The improvements in cellular energy may be mediated by the downstream AMPK signaling target PGC-1α, a transcriptional co-activator that stimulates the transcription factor nuclear respiratory factor1 (Nrf1) to induce expression of mitochondrial complex genes central to mitochondrial biogenesis [34]. Thus, the improved cellular energy may result from increased mitochondrial biogenesis and a higher oxidative capacity, either alone or in combination with the upregulation of AMPK-regulated catabolic (ATP-producing) pathways such as those involved in glucose and fatty acid metabolism. PT1’s effect on decreasing ROS may be explained by the observed upregulation of antioxidant enzymes catalase and SOD2. In addition to regulating *CAT* and *SOD2* gene expression, AMPK also controls the nuclear factor E2-related factor 2 (Nrf2)-antioxidant response element signaling pathway, which controls the expression of additional genes involved in detoxifying and eliminating reactive oxidants. Together, mechanisms involving increased antioxidant capacity through upregulated gene expression and increased oxidative phosphorylation through mitochondrial biogenesis may combine to improve overall cellular energy and redox status. Further testing examining mitochondrial content, and changes in glucose and fatty acid levels may help clarify the relative roles of each.

Finally, the metabolic and cellular effects of PT1 *in vitro* translated to significant effects at the whole system level. Mice injected with sodium iodate (SI) serve as a well-established model of retinal degeneration due to underlying mitochondrial dysfunction and oxidative stress [31, 32]. In targeting this mitochondrial phenotype, PT1 treatment improved both structural and functional signs of retinal degeneration, including restoring ERG values to normal levels. Additional studies are needed to determine if post-injury treatment is similarly efficacious, but given AMPK’s role in preventing cell death, it is expected to protect against further retinal damage caused by the SI insult. These studies suggest that the therapeutic strategy of AMPK activation may be applicable not only to mitochondrial disease, but a broad spectrum of disorders with secondary mitochondrial dysfunction including age-related macular degeneration. Additional *in vivo* efficacy studies using diverse mouse models of ETC complex defects are required to further evaluate PT1’s therapeutic potential in the treatment of disorders with mitochondrial dysfunction. Furthermore, while we did not observe any overt deleterious effects with PT1 treatment, *in vivo* safety studies will be required to determine any negative consequences associated with long-term AMPK activation.

Taken together, these studies demonstrate that AMP-independent activation of AMPK is more effective than mechanisms in which rising AMP levels regulate AMPK activity, possibly due to AMP-mediated pleiotropic effects. Through a mechanism of AMP-independent activation, the AMPK agonist PT1 was effective in ameliorating both the energy depletion and oxidative stress associated with mitochondrial dysfunction in and strongly support the therapeutic potential of PT1 in the treatment of mitochondrial disease and other mitochondrial-related disorders.

## References

[1] Herzig S, Shaw RJ. AMPK: guardian of metabolism and mitochondrial homeostasis. Nat Rev Mol Cell Biol. 2018 2;19(2):121–135. 10.1038/nrm.2017.95 28974774 PMC5780224

[2] Chinnery P. Mitochondrial Disorders Overview. GeneReviews updated 2014 [Internet].

[3] Koopman WJH, Distelmaier F, Smeitink JAM, Willems PHGM. OXPHOS mutations and neurodegeneration. EMBO J. 2013 1 9;32(1):9–29. 10.1038/emboj.2012.300 23149385 PMC3545297

[4] Jia H, Li X, Gao H, Feng Z, Li X, Zhao L, et al High doses of nicotinamide prevent oxidative mitochondrial dysfunction in a cellular model and improve motor deficit in a Drosophila model of Parkinson's disease. J Neurosci Res. 2008 7;86(9):2083–90. 10.1002/jnr.21650 18381761

[5] Atamna H.; Nguyen A.; Schultz C.; Boyle K.; Newberry J.; Kato H.; et al Methylene Blue Delays Cellular Senescence and Enhances Key Mitochondrial Biochemical Pathways. FASEB J. 2008 22(3): 703–712. 10.1096/fj.07-9610com 17928358

[6] Wright DJ, Renoir T, Smith ZM, Frazier AE, Francis PS, Thorburn DR, et al N-Acetylcysteine improves mitochondrial function and ameliorates behavioral deficits in the R6/1 mouse model of Huntington's disease. Transl Psychiatry. 2015 1 6;5 10.1038/tp.2014.131 PMC4312826 25562842

[7] Bavarsad Shahripour R, Harrigan MR, Alexandrov AV. N-acetylcysteine (NAC) in neurological disorders: mechanisms of action and therapeutic opportunities. Brain Behav. 2014 3;4(2):108–22. Mol Genet Metab. 2016 Nov; 119(3): 187–206. 10.1002/brb3.208 24683506 PMC3967529

[8] Douiev L, Soiferman D, Alban C, Saada A. The Effects of Ascorbate, N-Acetylcysteine, and Resveratrol on Fibroblasts from Patients with Mitochondrial Disorders. J Clin Med. 2016 12 22;6(1). 10.3390/jcm6010001 PMC5294954 28025489

[9] Pfeffer G, Majamaa K, Turnbull DM, Thorburn D, Chinnery PF. Treatment for mitochondrial disorders. Cochrane Database Syst Rev. 2012 4 18;4 10.1002/14651858.CD004426.pub3 PMC7201312 22513923

[10] El-Hattab AW, Zarante AM, Almannai M, Scaglia F. Therapies for mitochondrial diseases and current clinical trials. Mol Genet Metab. 2017 11;122(3):1–9. 10.1016/j.ymgme.2017.09.009 28943110 PMC5773113

[11] Camp KM, Krotoski D, Parisi MA, Gwinn KA, Cohen BH, Cox CS, et al Nutritional Interventions in Primary Mitochondrial Disorders: Developing an Evidence Base. Genet Metab. 2016 11;119(3):187–206. 10.1016/j.ymgme.2016.09.002 PMC5083179 27665271

[12] Viscomi C, Bottani E, Civiletto G, Cerutti R, Moggio M, Fagiolari G, et al In vivo correction of COX deficiency by activation of the AMPK/PGC-1α axis. Cell Metab. 2011 7 6;14(1):80–90. 10.1016/j.cmet.2011.04.011 21723506 PMC3130927

[13] Enns GM and Cowan TM. Glutathione as a Redox Biomarker in Mitochondrial Disease-Implications for Therapy. J Clin Med. 2017 5 3;6(5):50. 10.3390/jcm6050050 PMC5447941 28467362

[14] Garcia D, Shaw RJ. AMPK: Mechanisms of Cellular Energy Sensing and Restoration of Metabolic Balance. Mol Cell. 2017 6 15;66(6):789–800. 10.1016/j.molcel.2017.05.032 28622524 PMC5553560

[15] Hardie DG, Ross FA, Hawley SA. AMP-activated protein kinase: a target for drugs both ancient and modern. Chem Biol. 2012 10 26;19(10):1222–36. 10.1016/j.chembiol.2012.08.019 23102217 PMC5722193

[16] Li X, Wang L, Zhou XE, Ke J, de Waal PW, Gu X, et al Structural basis of AMPK regulation by adenine nucleotides and glycogen. Cell Res. 2015 1;25(1):50–66. 10.1038/cr.2014.150 25412657 PMC4650587

[17] Lan F, Weikel KA, Cacicedo JM, Ido Y. Resveratrol-Induced AMP-Activated Protein Kinase Activation Is Cell-Type Dependent: Lessons from Basic Research for Clinical Application. Nutrients. 2017 7 14;9(7). 10.3390/nu9070751 PMC5537865 28708087

[18] Golubitzky A, Dan P, Weissman S, Link G, Wikstrom JD, Saada A. Screening for active small molecules in mitochondrial complex I deficient patient's fibroblasts, reveals AICAR as the most beneficial compound. PLoS One. 2011;6(10). 10.1371/journal.pone.0026883 PMC3202581 22046392

[19] Lopes-Costa A, Le Bachelier C, Mathieu L, Rotig A, Boneh A, De Lonlay P, et al Beneficial effects of resveratrol on respiratory chain defects in patients' fibroblasts involve estrogen receptor and estrogen-related receptor alpha signaling. Hum Mol Genet. 2014 4 15;23(8):2106–19. 10.1093/hmg/ddt603 24365713

[20] Peralta S, Garcia S, Yin HY, Arguello T, Diaz F, Moraes CT. Sustained AMPK activation improves muscle function in a mitochondrial myopathy mouse model by promoting muscle fiber regeneration. Hum Mol Genet. 2016 8 1;25(15):3178–3191. 10.1093/hmg/ddw167 27288451 PMC5179920

[21] Goodyear L.J. The exercise pill—too good to be true? N. Engl. J. Med., 359 (2008), pp. 1842–1844. 18946072 10.1056/NEJMcibr0806723

[22] Coughlan KA, Valentine RJ, Ruderman NB, and Saha AK. AMPK activation: a therapeutic target for type 2 diabetes? Diabetes Metab Syndr Obes 2014; 7: 241–253. 10.2147/DMSO.S43731 25018645 PMC4075959

[23] Pang T, Zhang ZS, Gu M, Qiu BY, Yu LF, Cao PR, et al Small molecule antagonizes autoinhibition and activates AMP-activated protein kinase in cells. J Biol Chem. 2008 6 6;283(23):16051–60. 10.1074/jbc.M710114200 18321858 PMC3259642

[24] Zhang LN, Xu L, Zhou HY, Wu LY, Li YY, Pang T, et al Novel small-molecule AMP-activated protein kinase allosteric activator with beneficial effects in db/db mice. PLoS One. 2013 8 20;8(8). 10.1371/journal.pone.0072092 PMC3748009 23977216

[25] Li YY, Yu LF, Zhang LN, Qiu BY, Su MB, Wu F, et al Novel small-molecule AMPK activator orally exerts beneficial effects on diabetic db/db mice. Toxicol Appl Pharmacol. 2013 12 1;273(2):325–34. 10.1016/j.taap.2013.09.006 24055643

[26] Yu LF, Li YY, Su MB, Zhang M, Zhang W, Zhang LN, et al Development of Novel Alkene Oxindole Derivatives As Orally Efficacious AMP-Activated Protein Kinase Activators. ACS Med Chem Lett. 2013 3 25;4(5):475–80. 10.1021/ml400028q 24900695 PMC4027511

[27] Cool B, Zinker B, Chiou W, Kifle L, Cao N, Perham M, et al Identification and characterization of a small molecule AMPK activator that treats key components of type 2 diabetes and the metabolic syndrome. Cell Metab. 2006 6;3(6):403–16. 10.1016/j.cmet.2006.05.005 16753576

[28] Sanders MJ, Ali ZS, Hegarty BD, Heath R, Snowden MA, Carling D. Defining the mechanism of activation of AMP-activated protein kinase by the small molecule A-769662, a member of the thienopyridone family. J Biol Chem. 2007 11 9;282(45):32539–48. 10.1074/jbc.M706543200 17728241

[29] Salt IP and Hardie GD. AMP-Activated Protein Kinase–A Ubiquitous Signalling Pathway with Key Roles in the Cardiovascular System. Circ Res. 2017 5 26; 120(11): 1825–1841. 10.1161/CIRCRESAHA.117.309633 28546359 PMC5447810

[30] Jensen B. Measuring Mitochondrial Defects: XF Bioenergetic Analysis Identifies Defects in Human Skin Fibroblasts. Genetic Engineering & Biotechnology News. 5 2014, 34(10): 19–19

[31] Franco LM, Zulliger R, Wolf-Schnurrbusch UE, Katagiri Y, Kaplan HJ, Wolf S, et al Decreased visual function after patchy loss of retinal pigment epithelium induced by low-dose sodium iodate. Invest Ophthalmol Vis Sci. 2009 8;50(8):4004–10. 10.1167/iovs.08-2898 19339739

[32] Redfern WS, Storey S, Tse K, Hussain Q, Maung KP, Valentin JP, et al Evaluation of a convenient method of assessing rodent visual function in safety pharmacology studies: effects of sodium iodate on visual acuity and retinal morphology in albino and pigmented rats and mice. J Pharmacol Toxicol Methods. 2011 Jan-Feb;63(1):102–14. 10.1016/j.vascn.2010.06.008 20619348

[33] Li J., Novel Dual-Function Small-Molecule AMPK Activator Ameliorates Metabolic Syndrome. diabetes. 2010. Amer Diabetes Assoc 1701 N Beauregard St, Alexandria, VA 22311–1717 USA.

[34] Bergeron R, Ren JM, Cadman KS, Moore IK, Perret P, Pypaert M, et al Chronic Activation of AMP Kinase Results in NRF-1 Activation and Mitochondrial Biogenesis. Am J Physiol Endocrinol Metab. 2001 12;281(6):E1340–6. 10.1152/ajpendo.2001.281.6.E1340 11701451

